# The Chimeric Chaoyang-Zika Vaccine Candidate Is Safe and Protective in Mice

**DOI:** 10.3390/vaccines12020215

**Published:** 2024-02-19

**Authors:** Hao-Long Dong, Zhi-Li Chen, Mei-Juan He, Jia-Zhen Cui, Hao Cheng, Qing-Yang Wang, Xiang-Hua Xiong, Gang Liu, Hui-Peng Chen

**Affiliations:** Academy of Military Medical Sciences, Beijing 100071, China

**Keywords:** flavivirus, Zika virus, vaccine candidate, mouse, Chaoyang virus

## Abstract

Zika virus (ZIKV) is an emerging flavivirus that causes congenital syndromes including microcephaly and fetal demise in pregnant women. No commercial vaccines against ZIKV are currently available. We previously generated a chimeric ZIKV (ChinZIKV) based on the Chaoyang virus (CYV) by replacing the prME protein of CYV with that of a contemporary ZIKV strain GZ01. Herein, we evaluated this vaccine candidate in a mouse model and showed that ChinZIKV was totally safe in both adult and suckling immunodeficient mice. No viral RNA was detected in the serum of mice inoculated with ChinZIKV. All of the mice inoculated with ChinZIKV survived, while mice inoculated with ZIKV succumbed to infection in 8 days. A single dose of ChinZIKV partially protected mice against lethal ZIKV challenge. In contrast, all the control PBS-immunized mice succumbed to infection after ZIKV challenge. Our results warrant further development of ChinZIKV as a vaccine candidate in clinical trials.

## 1. Introduction

Zoonotic RNA virus outbreaks regularly arise in our connected world, mainly because of their high mutation rate and persistence in reservoir hosts [1,2,3]. ZIKV is an emerging member of the Flavivirus genus and is closely related to other medically important viral agents such as Japanese encephalitis virus, dengue virus, West Nile virus, and yellow fever virus. After its first isolation in Uganda in 1947, ZIKV rarely infected and caused diseases in humans until its outbreak in 2007 in Yap Island, Federated States of Micronesia, and its outbreak in 2013 in French Polynesia [1,2,4]. The ZIKV disease was characterized by fever, rash, arthralgia, and conjunctivitis in these outbreaks [5,6,7]. However, Brazil saw the worst outbreak of ZIKV in 2015, where the number of microcephaly cases in newborns and other birth defects in fetuses including intrauterine growth restriction, fetal demise, and cerebral calcifications increased sharply [8,9,10,11]. In 2016, the World Health Organization declared the ZIKV outbreak in Brazil a Public Health Emergency of International Concern for its association with newborn microcephaly [12]. The causal link between ZIKV infection in some adults and Guillain–Barré syndrome, an autoimmune disorder, where peripheral neurons and glial cells are damaged, has been established by a case–control study [2]. Compared with the other pathogenic flaviviruses, ZIKV attacked more organs and caused more severe infection outcomes such as testis damage, ocular defect, olfactory disorder, microcephaly, and fetal demise [10,13,14,15,16,17]. In addition, ZIKV can be transmitted from human to human via sexual transmission and blood transfusion [18]. Unlike the other pathogenic flaviviruses, ZIKV infection causes the most devastating outcomes in pregnant women [10,11,17,19,20]. Thus, safety is the top priority in the development of vaccines against ZIKV. Although, no large outbreak of ZIKV has been documented since 2016, the scientific community should continue to try to develop ZIKV vaccines for the next outbreak. ZIKV vaccine candidates based on various platforms such as DNA, mRNA, inactivated virus, and live attenuated virus have been developed in response to ZIKV outbreaks, and some of them have progressed to clinical trials [21,22,23,24,25].

Arthropod-borne flaviviruses have wreaked havoc on public health for over a century and continue to do so [26]. Vaccines have been proven to be effective in the control of pathogenic flaviviruses such as yellow fever virus and Japanese encephalitis virus [27]. However, even with the help of the most successful vaccine yellow fever virus 17D strain, sporadic outbreaks of yellow fever keep arising because of spillover from animal hosts to humans [28,29]. Furthermore, vaccine-associated viscerotropic disease and neurotropic disease caused by the 17D vaccine strain in rare vaccines remains an unresolved safety issue [30]. Fractional-dose yellow fever vaccination was proposed and accepted because of vaccine shortage in the case of yellow fever outbreak in the Democratic Republic of Congo [31]. Supply issues of the 17D vaccine also urgently need to be addressed. The widespread use of Japanese encephalitis vaccines derived from genotype III partly drove the shift of the dominant genotype from genotype III to genotype I, reducing the efficacy of the vaccines in use [32,33,34,35,36,37,38,39]. Although vaccines against pathogenic flaviviruses such as yellow fever and Japanese encephalitis vaccines have been very successful in reducing the number of infections, they have failed to eradicate viruses like smallpox and poliovirus types 2 and 3 [28,40,41,42,43,44]. Despite the success of vaccines, these viruses continue to pose a constant threat to public health mainly because of their persistence in wildlife reservoir hosts and transmission via mosquito bites [36,45,46]. The wildlife reservoir hosts such as monkeys and water birds cannot be vaccinated like humans. Transmission via mosquito bites is more difficult to cut off than other normal routes such as the fecal oral route and sexual contact. Vaccination of the susceptible population and the use of insecticide and bed nets only can reduce the number of infections and hospitalizations caused by pathogenic flaviviruses. Outbreaks of flavivirus infection keep reemerging as the population’s immunity wanes, and new variants of viruses emerge. To date, all the vaccines against flaviviruses ever developed have been intended for use in humans and domestic animals. Thus, novel, safe, and efficacious vaccines are urgently needed to eliminate the pathogenic flaviviruses from both their wildlife hosts and insect vectors once and for all.

Flaviviruses have a single positive-strand RNA genome of approximately 11 kb with a single open reading frame encoding a polyprotein processed by viral and cellular proteases into three structural proteins, designated as capsid ©, premembrane (prM), and envelope (E), and seven non-structural proteins. The premembrane is further cleaved into the functional membrane by the host enzyme furin [47]. The E protein is responsible for receptor binding and the main antigen for the development of neutralizing antibodies. Flavivirus chimeras expressing prM-E proteins of the pathogenic flaviviruses have been explored as vaccine candidates, and some of them are licensed [48,49,50,51,52]. The pathogenic flaviviruses are primarily transmitted between vertebrates by mosquito or tick bites, and humans are occasional hosts. However, there is a group of flaviviruses isolated in the past decades that is not able to infect vertebrates, and thus, these viruses are referred to as insect-specific flaviviruses [53,54,55,56,57,58]. Some insect flaviviruses have been used as the backbone for constructing chimeric flavivirus vaccine candidates for their inability to infect vertebrates [48,59]. The concept of vaccination via mosquito bites was even proposed for a ZIKV vaccine candidate using an insect-specific flavivirus backbone [59]. One of the insect-specific flaviviruses, CYV was initially isolated in 2008 in Liaoning province, China [58]. We have previously generated ChinZIKV based on CYV by replacing the prME protein of CYV with that of a clinical isolate of ZIKV GZ01 [60]. Herein, we evaluated the safety and immunogenicity of ChinZIKV as a vaccine candidate against ZIKV in a type I interferon receptor knockout (IFNAR−/−) mouse model [61]. Our results demonstrate that ChinZIKV is safe in both adult and suckling mice and elicits partial protective immunity after a single immunization. As a ZIKV vaccine candidate based on an insect-specific flavivirus, ChinZIKV has the potential to eliminate ZIKV from its wildlife reservoir hosts.

## 2. Materials and Methods

### 2.1. Cells and Viruses

African green monkey kidney Vero cells (ATCC, CCL-81) were maintained at 37 °C and 5% CO_2_ in a high-glucose Dulbecco’s modified Eagle’s medium (DMEM), while the Aedes albopictus C6/36 cells (ATCC, CRL-1660) were maintained at 28 °C in RPMI1640 Medium (Gibco, Waltham, MA, USA). Both mediums were supplemented with 10% fetal bovine serum (FBS, Gibco, Waltham, MA, USA), 10 mM 4-(2-hydroxyethyl)-1-piperazineethanesulfonic acid (HEPES, Gibco, Waltham, MA, USA), and 1% penicillin streptomycin (P/S, Gibco, USA). The ZIKV GZ01 strain and ChinZIKV were described in previous works [60,62,63]. Briefly, the GZ01 strain of ZIKV was isolated from the urine sample of a ZIKV-infected patient. ChinZIKV was obtained by replacing CYV prM-E with that of the ZIKV GZ01 strain. ZIKV and ChinZIKV were grown and passaged in C6/36 cells. Viral titers of stocks were determined by a modified focus-forming assay [59]. Briefly, 10-fold dilutions of virus supernatants were seeded on confluent C6/36 cells in a 48-well plate for 1 h. Then, the supernatants were replaced with maintenance medium supplemented with 20 mM NH_4_Cl, which was used to inhibit the secondary infection of flaviviruses. Indirect immunofluorescence assay was performed on the cells after incubation at 28 °C for 3 days, as previously described [60]. Virus foci were stained with a mouse monoclonal antibody (1:500 diluted) (GeneTex, Irvine, CA, USA, Cat No. GTX57154) against the flavivirus envelope protein followed by goat anti-mouse IgG conjugated with Alexa Fluor 488 (1:500 diluted) (ThermoFisher Scientific, Waltham, MA, USA). Cell nuclei were visualized by DAPI staining (Solarbio, Beijing, China), and virus foci were counted under a fluorescence microscope (Axio Observer, Zeiss, Oberkochen, Germany).

### 2.2. Animal Experiments

All animal experiments were performed following the guidelines of the Chinese Regulations of Laboratory Animals and Laboratory Animal-Requirements of Environment and Housing Facilities. All procedures were approved by the Animal Experiment Committee of the Laboratory Animal Center, Academy of Military Medical Sciences (AMMS), China (IACUC-DWZX-2023-007). Specific-pathogen-free *ifnar*-deficient mice in C57BL/6 background (IFNAR−/− C57BL/6 mice) were purchased from Beijing HuaFuKang and housed within the vivarium of AMMS. Each inoculation group was separately housed in rodent cages under biosafety level 2 (BSL-2) conditions.

Experiment 1: evaluation of the safety of ChinZIKV in IFNAR−/− C57BL/6 mice. Four-week-old specific-pathogen-free female IFNAR−/− C57BL/6 mice were randomly divided into 2 groups. One group of mice was subcutaneously inoculated with 10^4^ FFU of the ZIKV GZ01 strain per mouse (*n* = 4). Another group of mice was subcutaneously inoculated with 10^4^ FFU of ChinZIKV (*n* = 6). The mice were evaluated daily for weight change, signs of disease, and mortality. Blood samples were collected successively for 5 days by bleeding the tail vein. Mice infected with ZIKV were euthanized before reaching the moribund state at 7 or 8 days post infection, and then the brains and spleens were harvested. The same number of mice infected with ChinZIKV were euthanized, and their organs were harvested as well.

Experiment 2: evaluation of the safety of ChinZIKV in IFNAR−/− C57BL/6 suckling mice. One-day-old specific-pathogen-free IFNAR−/− C57BL/6 suckling mice were randomly divided into 2 groups (*n* = 5). Two groups of suckling mice were intracranially inoculated with 10^2^ FFU of the ZIKV GZ01 strain and ChinZIKV per mouse, respectively. These two groups of suckling mice were monitored daily for weight change and mortality.

Experiment 3: evaluation of the immunogenicity of ChinZIKV in IFNAR−/− C57BL/6 mice. Four-week-old specific-pathogen-free female IFNAR−/− C57BL/6 mice were randomly divided into 2 groups. One group of mice was subcutaneously inoculated with 104 FFU of ChinZIKV (*n* = 11), five of which were kept alive for weight change monitoring and evaluation of the symptom of ZIKV infection, whereas the remainder were euthanized to evaluate the viral loads in the brains and spleens. All the ChinZIKV-immunized mice were tested for viremia. Another group of mice was used as the control with subcutaneous administration of equal volume of PBS (*n* = 7). Blood samples were reserved at 28 days post ChinZIKV immunization, and then all mice were challenged with 104 FFU of the ZIKV GZ01 strain. The weight change, signs of disease, and mortality of the mice were evaluated each day, and blood samples were collected at 2 and 3 days after ZIKV challenge. Mice monitoring, blood sample collection, and organ harvest were performed similar to Experiment 1.

### 2.3. Measurement of Viral Genome by qRT-PCR

The brains and spleens were homogenized using a bead beater instrument (MagNA Lyser, Roche, Shanghai, China). Viral RNA in the blood samples and homogenates of organ samples was quantified by qRT-PCR with M5 HiPer Direct Viral RNA qPCR kit (Mei5 Biotechnology, Beijing, China), according to the manufacturer’s instructions. TaqMan one-step quantitative reverse transcriptase PCR (qRT-PCR) was performed in a real-time PCR system (Quantstudio 3, ThermoFisher Scientific, Waltham, MA, USA) using standard cycling conditions with primers and the probe set targeting the ZIKV E gene (forward, 5′-CCGCTGCCCAACACAAG-3′; probe, 5′-(FAM)AgCCTACCTTgACAAgCA(A/g)TCAgACACTCAA(BHQ1)-3′; reverse, 5′-CCACTAACGTTCTTTTGCAGACAT-3′). Viral loads were calculated based on a standard curve produced using serial 10-fold dilutions of extracted viral RNA (Purelink RNA mini kit, ThermoFisher Scientific, Waltham, MA, USA) and expressed on a log_10_ scale as viral RNA copies per gram or per milliliter.

### 2.4. Neutralizing Antibody Response Determination

Mice sera were collected from blood samples by centrifugation at 4 °C. After heat inactivation, the sera were titrated using 4-fold dilutions on Vero cells. Serum neutralization assays were performed by incubating serial dilutions of heat-inactivated sera (in duplicate, 50 µL/well on a 96-well plate) with 10^4^ FFU of ZIKV (50 µL/well) for 1 h before they were added to Vero cells (10^4^ in 100 µL/well). After a 5-day incubation at 37 °C and 5% CO_2_, the plates were fixed with formaldehyde and stained with 0.1% crystal violet for 30 min. After washing and drying the plates, 100 µL of cold methanol was added per well, and the plate was measured at optical density (OD) at 595 nm in a multimode plate reader (Enspire, Revvity, Shanghai, China). The reciprocal 50% neutralizing titer was determined by linear interpolation of the OD values using Prism 8.0.2 (GraphPad, San Diego, CA, USA) with 0% neutralization set for the wells with virus only, whereas 100% neutralization was set for the wells without virus and anti-serum.

### 2.5. Data Analysis

GraphPad Prism 8.0.2 was used for data analysis. Weight change was compared using two-way ANOVA. Kaplan–Meier survival curves were analyzed by the log rank test. For viral burden analysis, the log titers and levels of viral RNA were analyzed by unpaired *t* test. A *p* value of <0.05 indicated statistically significant differences.

## 3. Results

### 3.1. ChinZIKV Is Safe in Immunodeficient Mice

**Figure 1:**
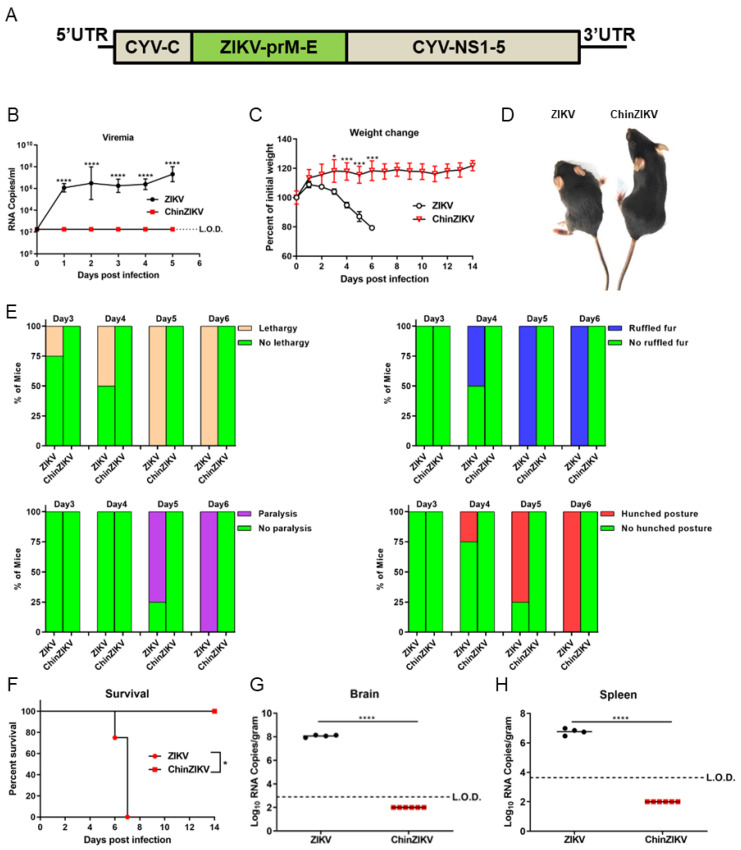
ChinZIKV does not infect or cause disease in IFNAR−/− C57BL/6 mice. (**A**) schematic diagram of the genome of ChinZIKV is shown. (B-H) Two groups of mice were subcutaneously inoculated with 10^4^ FFU ZIKV (*n* = 4) or ChinZIKV (*n* = 6), respectively. The dashed lines indicate the limit of detection. (**B**) Viral RNA in serum was determined by qRT-PCR. Data were analyzed by two-way ANOVA. Data are presented as means ± standard deviations. **** *p* < 0.0001. (**C**) Mice were weighed daily, and weights are expressed as percentage of the initial body weight. Data were analyzed by two-way ANOVA. Data are presented as means ±standard deviations. * *p* < 0.05, *** *p* < 0.001. (**D**) Representative mice from the two groups with or without disease signs. (**E**) The percentage of mice infected with ZIKV or ChinZIKV displaying the indicated signs at the indicated time points is presented. (**F**) Mortality in the two groups of mice was monitored for 14 days. Data were analyzed by log-rank (Mantel–Cox) test. * *p* < 0.05. (**G**,**H**) Moribund ZIKV-infected mice or healthy mice inoculated with ChinZIKV were euthanized at 6 or 7 dpi. Viral loads in brains and spleens were determined by qRT-PCR. Data were analyzed by unpaired *t* test. **** *p* < 0.0001.

Previously, we generated ChinZIKV by replacing the prM-E of CYV with that of ZIKV GZ01 and established that ChinZIKV did not replicate in Vero and BHK-21 cell lines, which were derived from vertebrates (Figure 1A) [60]. However, it is unknown whether ChinZIKV infects and causes disease in mice. To address this issue and evaluate the safety of ChinZIKV in mice, we chose a well-established IFNAR−/− C57BL/6 mouse model of ZIKV infection that recapitulated many features of infection and disease in humans. Two groups of four-week-old IFNAR−/− C57BL/6 mice were subcutaneously administered 10^4^ FFU of ChinZIKV or ZIKV, respectively. ZIKV infected, replicated, and led to death in IFNAR−/− C57BL/6 mice, whereas ChinZIKV did not establish infection in mice (Figure 1A,E). Mice infected with ZIKV developed viremia at 1 day post inoculation (dpi), whereas no viral RNA was detected in mice inoculated with ChinZIKV from 1 dpi to 5 dpi. Viremia in mice infected with ZIKV reached 10^7^ viral RNA copies/mL at 5 dpi (Figure 1B). ZIKV-infected mice began to lose weight at 2 dpi, with ~20% of the starting weight lost by 6 dpi. The ChinZIKV inoculation group of mice did not lose weight at all and gained about 20% body weight 14 days after inoculation. The difference between the body weight of the two groups of mice became statistically significant at 3 dpi (Figure 1C). Lethargy was observed in ZIKV-infected mice as early as 3 dpi, and clinical signs of ZIKV infection, including ruffled fur, paralysis, and hunched posture, followed (Figure 1D,E). Consistent with the measurement of viremia by qRT-PCR, ChinZIKV-infected mice did not show any signs of infection (Figure 1E). By 8 dpi, all the ZIKV-infected mice succumbed to infection, with a median survival time of 7 days. All the ChinZIKV group of mice survived (Figure 1F). To further characterize the safety of ChinZIKV in mice, we euthanized moribund mice infected with ZIKV and healthy mice inoculated with ChinZIKV at 6 or 7 dpi. Viral loads were determined by qRT-PCR in the brains and spleens harvested from the euthanized mice in the two groups. No viral RNA was detected in the brains and spleens of ChinZIKV-infected mice. In contrast, the viral loads in ZIKV-infected mice reached 10^8^ and 10^7^ viral genome copies/mL in brains and spleens, respectively (Figure 1G,H). Taken together, these experiments demonstrate that ChinZIKV does not infect mice and is completely safe.

### 3.2. Safety of ChinZIKV in Suckling Mice

**Figure 2:**
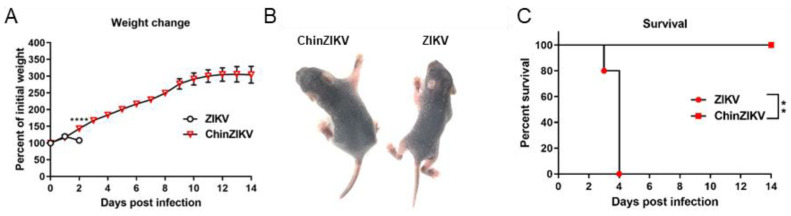
ChinZIKV is safe even in IFNAR−/− C57BL/6 suckling mice. (**A**–**C**) Two groups of IFNAR−/− C57BL/6 suckling mice were intracranially inoculated with 1000 FFU ZIKV or ChinZIKV, respectively (*n* = 5). (**A**) Mice were weighed daily, and weights are expressed as percentage of the initial body weight. Data were analyzed by two-way ANOVA. Data are presented as means ± standard deviations. **** *p* < 0.0001. (**B**) Representative mice from the two groups with or without disease signs. (**C**) Mortality in the two groups of mice was monitored for 14 days. Data were analyzed by log-rank (Mantel–Cox) test. ** *p* < 0.01.

To determine whether ChinZIKV is safe in suckling mice, who are more susceptible to ZIKV infection, we further evaluated the safety of ChinZIKV in suckling mice. We intracranially inoculated two groups of one-day-old suckling IFNAR−/− C57BL/6 mice with 10^2^ FFU of ZIKV or ChinZIKV, respectively. Weight change, morbidity, and mortality were monitored daily. ZIKV-infected suckling mice began to lose weight and succumbed to infection at 2 dpi. In contrast, the body weight of ChinZIKV-infected suckling mice trebled at 14 dpi, and no signs of infection or mortality were observed in this group of mice (Figure 2A–C). These results indicate ChinZIKV is safe even in immunodeficient suckling mice.

### 3.3. Immunogenicity and Efficacy of ChinZIKV

**Figure 3:**
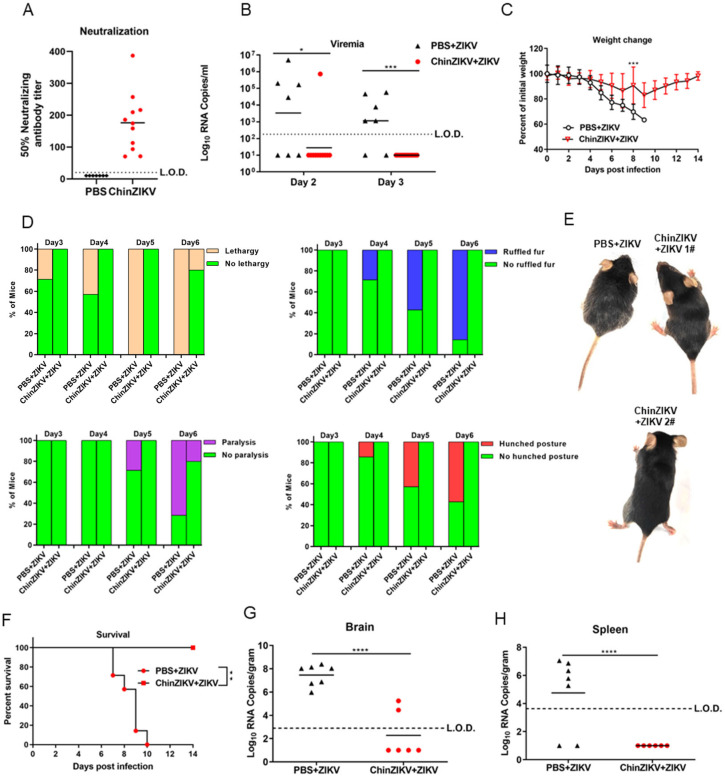
A single dose of ChinZIKV elicited partial protective immunity in IFNAR−/− C57BL/6 mice. (**A**–**H**) Two groups of mice were subcutaneously inoculated with 10^4^ FFU ChinZIKV or PBS, respectively (*n* = 7 for PBS group, *n* = 11 for ChinZIKV group). The dashed lines indicate the limit of detection. (**A**) Neutralizing antibody titers in serum were determined 28 days post immunization (*n* = 7 for PBS group, *n* = 11 for ChinZIKV group). (**B**) Immunized mice were challenged with 10^4^ FFU ZIKV via subcutaneous route 30 days post immunization. Viral RNA in serum was determined by qRT-PCR at day 2 or day 3 post challenge (*n* = 7 for PBS group, *n* = 11 for ChinZIKV group). Data were analyzed by unpaired *t* test. * *p* < 0.05, **** *p* < 0.0001. (**C**) Mice were weighed daily, and weights are expressed as percentage of the initial body weight (*n* = 7 for PBS group, *n* = 5 for ChinZIKV group). Data were analyzed by two-way ANOVA. Data are presented as means ±standard deviations. *** *p* < 0.001. (**D**) The percentage of mice challenged with ZIKV displaying the indicated signs at the indicated time points is presented. (*n* = 7 for PBS + ZIKV group, *n* = 5 for ChinZIKV + ZIKV group). (**E**) Representative mice from the two groups with or without disease signs. ChinZIKV + ZIKV 1# mouse exhibited no disease signs. ChinZIKV + ZIKV 2# exhibited hindlimb paralysis. (**F**) Mortality in the two groups of mice was monitored for 14 days. Data were analyzed by log-rank (Mantel–Cox) test. ** *p* < 0.01. (*n* = 7 for PBS + ZIKV group, *n* = 5 for ChinZIKV + ZIKV group). (**G**,**H**) Moribund PBS-immunized mice or healthy mice immunized with ChinZIKV were euthanized at 7–10 dpi. Viral loads in brains and spleens were determined by qRT-PCR. Data were analyzed by unpaired *t* test. **** *p* < 0.0001. (*n* = 7 for PBS + ZIKV group, *n* = 6 for ChinZIKV + ZIKV group).

Next, we evaluated the immunogenicity and efficacy of ChinZIKV in IFNAR−/− C57BL/6 mice. Two groups of four-week-old female IFNAR−/− C57BL/6 mice were subcutaneously inoculated with 10^4^ FFU ChinZIKV or an equal volume of PBS (*n* = 7–11). Neutralizing antibody titers were determined 28 days post immunization. Inoculation with ChinZIKV elicited neutralizing antibody titers of 70–380 dilution folds (Figure 3A). After challenge with 10^4^ FFU ZIKV via subcutaneous route at 30 days post immunization, only one of eleven ChinZIKV-immunized mice developed viremia, as ZIKV RNA was detected by qRT-PCR in serum sample. In contrast, five of seven mice immunized with PBS developed viremia by 3 days post challenge (Figure 3B). Although both groups of mice began to lose weight 2 days after challenge, the mice immunized with PBS continued to lose weight sharply, and the difference in the weight between the two groups became statistically significant 8 days after challenge (Figure 3C). Furthermore, the whole group of mice immunized with ChinZIKV completely recovered from weight loss (Figure 3C). Lethargy was observed as early as 3 days post challenge in mice immunized with PBS, and all the mice showed at least one symptom of infection at 6 days post challenge. Notably, one of the mice immunized with ChinZIKV exhibited lethargy and paralysis too, but this mouse completely recovered from ZIKV infection at 14 days post challenge (Figure 3D,E). All the control PBS-immunized mice succumbed to ZIKV infection 10 days after ZIKV challenge, whereas none of the ChinZIKV vaccinated mice became moribund (Figure 3F). ZIKV RNA was detected in all the brains harvested from the euthanized mice immunized with PBS compared with only two of six mice immunized with ChinZIKV. No ZIKV RNA was detected in the spleens of ChinZIKV immunized mice, whereas five of seven in the control group were positive for ZIKV RNA. These results demonstrate that a single dose of ChinZIKV reduced disease manifestations, viremia, viral loads in brains and spleens, and virus dissemination via the blood–brain barrier.

## 4. Discussion

Safe and efficacious vaccines are the most effective measures against arboviral flaviviruses in the absence of specific antivirals, proven by the success of yellow fever, Japanese encephalitis, and tick-borne encephalitis vaccines [27,64]. Unlike the abovementioned flaviviruses, ZIKV causes congenital malformation in fetuses of pregnant women [8,9,11]. The safety requirement should be prioritized when developing ZIKV vaccine candidates. Herein, we illustrate the safety, immunogenicity, and efficacy of ChinZIKV as a vaccine candidate against ZIKV. ChinZIKV did not infect and replicate in mice, as its backbone was derived from an insect-specific flavivirus. We proved the safety of ChinZIKV in four-week-old female mice. No viral genome of ChinZIKV was detected in the blood, brains, or spleens of the inoculated ifnar-deficient mice in C57BL/6 background (Figure 1B,G,H). No morbidity or mortality was observed in this group of mice inoculated with ChinZIKV (Figure 1D–F). Since mice are susceptible to flaviviruses in an age-dependent manner [65,66], to determine whether the safety of ChinZIKV extends to suckling mice, we further demonstrated the safety of ChinZIKV in one-day-old ifnar-deficient suckling mice that are very susceptible to flavivirus infection [65]. ChinZIKV did not cause any clinical presentations or demise even in ifnar-deficient suckling mice (Figure 2A–C). The use of insect-specific flavivirus as the genetic backbone provides ChinZIKV with a safety advantage over other live attenuated vaccine candidates and makes it more appropriate for use in pregnant women.

Most vaccine platforms have to balance between safety and immunogenicity. To some degree, the safety advantage of ChinZIKV compromised its immunogenicity. A single dose of ChinZIKV induced partial protective immune responses. The mice vaccinated with ChinZIKV experienced weight loss after ZIKV challenge (Figure 3C). Notably, one of the mice vaccinated with ChinZIKV developed viremia and exhibited hindlimb paralysis, and ZIKV RNA was detected in the brains of two mice in the group (Figure 3B,D,E,G). This breakthrough infection might be attributed to the low dose of ChinZIKV and its non-replicative feature. Unfortunately, we could not examine whether the breakthrough infections were due to low levels of neutralizing antibodies because the vaccinated mice were treated as a group and not traced individually. However, these mice regained body weight and recovered from ZIKV infection. It is possible that a prime-boost vaccine strategy or the use of an adjuvant might elicit more robust immune responses. Not all of the PBS immunized mice developed viremia at the indicated time points after ZIKV challenge possibly because this group of mice were about 8 weeks old by the time of challenge (Figure 3B) or their viremia was delayed.

There are some limitations in our study. Limited types of cell lines derived from vertebrates were tested for ChinZIKV [60]. More cell types from humans are needed to fully characterize the host tropism and safety of ChinZIKV. Competition between ChinZIKV and ZIKV within mosquitoes are needed to evaluate the potential of ChinZIKV as a mosquito-delivered vaccine candidate. Both humoral and cellular immune responses are involved in protection against viral infection. Evaluation of the cellular immune responses and functional non-neutralizing antibodies elicited by ChinZIKV was lacking in this study. Comparison of the immunity levels before and after ZIKV challenge in vaccinated mice should improve the understanding of the interaction between vaccine-induced immunity and the challenge virus and inform the prime-boost decision process. Further studies on the safety and immunogenicity of ChinZIKV in immunocompetent mice and non-human primates should bridge these knowledge gaps.

The chimeric vaccine candidates based on an insect-specific flavivirus developed by us and other groups represent a novel type of vaccine, which has the potential to eliminate mosquito-borne pathogenic flaviviruses once and for all [67]. Wen et al. have constructed a chimeric flavivirus by replacing the CYV prM-E with that of a ZIKV African lineage strain MR766 [59]. They successfully infected mosquitoes by feeding them with blood containing chimeric virus and vaccinated mice via mosquito bites. The mice were protected against ZIKV challenge by this mosquito-delivered vaccine. Instead of elimination of mosquito vectors to control mosquito-borne flaviviruses, these insects may be used against the pathogenic flaviviruses. Introduction of chimeric vaccines based on insect-specific flaviviruses into wild mosquito vectors could possibly facilitate the elimination of the pathogenic flaviviruses in their reservoir hosts [59]. Wildlife hosts can be easily vaccinated by being bitten by mosquitoes artificially infected with chimeric vaccines like ChinZIKV via intrathoracic injection and blood meal [59,68,69]. Moreover, the pathogenic flaviviruses could possibly be driven out of the entire mosquito vector population by more competitive chimeric flaviviruses based on insect specific flaviviruses. The mechanisms of flavivirus phenotypes have been intensively studied, and some mutations in specific sites in the flavivirus genomes could change the viral fitness, virulence, tropism, and transmission route and the ability to evade host immune responses [70,71,72,73]. To select the most appropriate insect-specific flavivirus backbone for the construction of chimeric flavivirus expressing structural proteins of pathogenic flaviviruses, more efforts should be put into the characterization of newly isolated insect-specific flaviviruses and their interaction with mosquito vectors. By bridging the knowledge gaps about insect-specific flaviviruses, we can rationally design and construct chimeric flaviviruses with increased fitness in comparison with the pathogenic flaviviruses. Combined with reverse genetics, insect-specific flaviviruses could play an important role in the eradication of the pathogenic flaviviruses. These ideas and technologies could even be useful in the combat against other vector-borne viruses. Ethics and biosafety issues need to be fully considered in the development and use of mosquito-delivered chimeric flavivirus vaccines. Right now, this concept is only in the stage of experiment in a few laboratories, but it warrants further research and development given its potential benefits.

## 5. Conclusions

In summary, we evaluated the safety and immunogenicity of ChinZIKV in immunodeficient mice. ChinZIKV is completely safe in both adult and suckling mice, because its parental CYV does not infect vertebrates including mice. A single dose of ChinZIKV administered via subcutaneous route partially protected mice against lethal ZIKV infection. The efficacy of ChinZIKV via the bite of infected mosquito needs to be assessed. Further studies are needed to compare the fitness of ChinZIKV and its parental ZIKV in mosquito vectors. Reverse genetics could contribute to enhance the fitness and transmission capacity of chimeric flaviviruses including ChinZIKV in reservoir hosts and mosquito vectors.

## Data Availability

The data presented in this study are available on request from the corresponding author.
